# Diagnostic Cytokines and Comparative Analysis Secreted from Exfoliated Deciduous Teeth, Dental Pulp, and Bone Marrow Derived Mesenchymal Stem Cells for Functional Cell-Based Therapy

**DOI:** 10.3390/ijms20235900

**Published:** 2019-11-24

**Authors:** Yoichi Yamada, Sayaka Nakamura-Yamada, Eri Umemura-Kubota, Shunsuke Baba

**Affiliations:** 1 Department of Oral Implantology, Osaka Dental University, 1-5-17 Otemae Chuo-ku, Osaka 540-0008, Japan; yamada-s@cc.osaka-dent.ac.jp (S.N.-Y.); baba-s@cc.osaka-dent.ac.jp (S.B.); 2 Department of Infectious Diseases and Applied Immunology, IMSUT Hospital of the Institute of Medical Science, The University of Tokyo, 4-6-1 Shiroganedai, Minato-ku, Tokyo 108-8639, Japan; 3 Department of Oral and Maxillofacial Surgery, Saisyukan Hospital, 111 Sikata Nishimuramae, Kitanagoya, Aichi 481-0004, Japan; erimakilondon@gmail.com

**Keywords:** stem cells from exfoliated deciduous teeth (SHED), dental pulp stem cells (DPSCs), bone marrow-derived mesenchymal stem cells (BMMSCs), cytokine expression, cell-based therapy, therapeutic paradigm shift

## Abstract

The aim of the study was to clarify the distinctive features of stem cells for effective cell-based therapy strategies in regenerative medicine. The expression levels of cytokines secreted from stem cells from exfoliated deciduous teeth (SHED), dental pulp stem cells (DPSCs), and bone marrow derived mesenchymal stem cells (BMMSCs) were examined to identify the details of their characteristics. A total of 174 cytokines were analyzed using cytokine antibody array, and their expression levels were confirmed by an enzyme-linked immunosorbent assay. These results indicated that 11 cytokines that were related to tissue regeneration, including growth factors, chemokines, and inflammatory cytokines, were identical in SHED, DPSCs, and BMMSCs. The comparative analyses between SHED and BMMSCs revealed that hepatocyte growth factor (HGF), matrix metalloproteinase-3, and stromal cell derived factor 1 (SDF-1) were expressed 6.7-, 2.5-, and 2.1-fold higher, respectively, in SHEDs. HGF was also expressed 3.4-fold higher in DPSCs than BMMSCs. Monocyte chemoattractant protein-1, and-3 were expressed more strongly in BMMSCs. SHED contained significantly higher SDF-1 levels than DPSCs. The distinct cytokine secretion indicated that they had different character besides basic MSC features. This knowledge of diagnostic cytokines analysis secreted from SHED, DPSCs, and BMMSCs extends our understanding, and can provide a novel therapeutic paradigm shift for functional cell-based therapy.

## 1. Introduction

**Figure 1:**
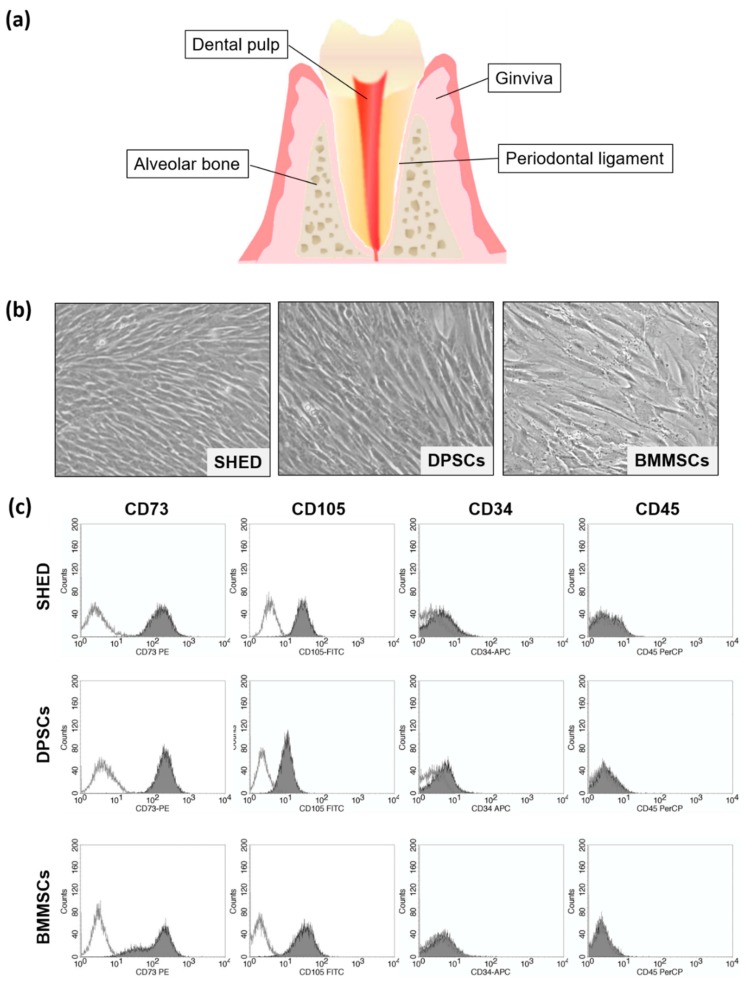
(**a**) Schema of tooth and periodontal tissue. (**b**) Cell morphology of stem cells from exfoliated deciduous teeth (SHED), dental pulp stem cells (DPSCs), and bone marrow-derived mesenchymal stem cells (BMMSCs). (×50) (**c**) Characteristics of SHED, DPSCs, and BMMSCs were analyzed using flow cytometry. Typical flow cytometric analysis diagrams on the expression of the mesenchymal stem/stromal cell (MSC) markers (CD73 and CD105), as well as of the endothelial/hematopoietic markers (CD34 and CD45).

Mesenchymal stem/stromal cells (MSCs) are multipotent stem cells that have been widely investigated as a powerful tool for tissue engineering and regenerative medicine. MSCs were first found in bone marrow [1] followed by various tissues such as oral tissue [2], adipose tissue [3], muscle, dermis [4], and fetal tissue [5]. Among them, stem cells from oral tissue including dental pulp, periodontal ligament, dental follicle, apical papilla, oral mucosa, and gingiva have a promising origin due to their easily isolated and accessible sources (Figure 1a).

Stem cells from exfoliated deciduous teeth (SHED) and dental pulp stem cells (DPSCs) were identified as MSCs that have the capacity of self-renewal and multilineage differentiation [6,7,8,9]. SHED and DPSCs have been widely studied because they have been reported to be useful not only for dental diseases but also for various systemic diseases including neurological diseases, circulatory diseases, internal diseases, eye diseases, bone diseases, and orthopedic disorders [2]. Although MSCs have a similar phenotype as defined by The International Society for Cellular Therapy [10], those from various tissues often exhibit different features [11]. In our previous studies, we performed comparative analyses of the gene profile between bone marrow-derived mesenchymal stem cells (BMMSCs) and DPSCs, SHED and DPSCs, and SHED and BMMSCs [12,13,14]. These results indicated that SHED, DPSCs and BMMSCs had additional identifying characteristics in addition to basic MSC features.

Recently, there have been extensive evaluations involving clinical trials using MSC-based therapies for treating various diseases [15]. Despite their therapeutic potential for clinical use, the precise mechanism of the effects remains an area of intensive investigation. It is also important to functionalize the circumstances and environment for the tissues and cells for clinical success. Previous studies indicated that the transplanted cells played multiple and important roles; they could not only migrate in their host tissues and participate directly in the regeneration of tissue, but also have paracrine effects [2,15,16]. For paracrine effects, MSCs produce many cytokines involved in various mechanisms including decreasing inflammation, enhancing progenitor cell proliferation, improving tissue repair, and decreasing infection [16]. Since MSCs have identical characteristics that reflect their parent sources, different kinds of cytokines can be released by the various cell sources of MSCs. More recently, MSC-derived extracellular vesicles are being examined for their role in MSC-based cellular therapy. These vesicles can also influence tissue responses to injury, infection, and disease and includes cytokines and growth factors, signal lipids, mRNAs, and regulatory miRNAs [16]. However, the variety of cytokines that are secreted from SHED, DPSCs, and BMMSCs remains unclear.

In this study, we performed the cytokine antibody array analysis to examine and compare the cytokines secreted from SHED, DPSCs, and BMMSCs to identify the details of their characteristics to further develop current cell-based therapy and cell drug discovery. The comparative cytokine profiles of SHED, DPSCs, and BMMSCs suggest a potential therapeutic implication for the choice of MSCs in regenerative medicine. The findings have direct clinical relevance. Although all three cell types have the characteristics of MSCs, the relative differences in the cytokine profiles has a potential implication for therapeutic regeneration as the choice of SHED, DPSCs, or BMMSCs for the best tissue repair may be made based on the type of tissue or the conditions under which the tissue regeneration is required. Studies such as the present study can be useful for exploiting the differences in tissue-specific stem cells for best therapeutic benefits. The aim of the study is to clarify and apply the distinctive features of stem cells for effective strategies for regenerative medicine.

## 2. Results

### 2.1. Characterization of SHED (Stem Cells from Exfoliated Deciduous Teeth), DPSCs (Dental Pulp Stem Cells), and BMMSCs (Bone Marrow-Derived Mesenchymal Stem Cells)

SHED, DPSCs, and BMMSCs are plastic-adherent and spindle-shaped cells (Figure 1b). To confirm the characteristics of SHED, DPSCs, and BMMSCs used in this study, flow cytometry analysis was performed. SHED, DPSCs, and BMMSCs were positive for the mesenchymal stem cell markers (CD73 and CD105), and negative for endothelial/hematopoietic markers (CD34 and CD45) (Figure 1c). These phenotypes were consistent with the criteria for the identification of MSCs defined by The International Society for Cellular Therapy [10].

### 2.2. Cytokines Secreted from SHED, DPSCs, and BMMSCs

**Figure 2:**
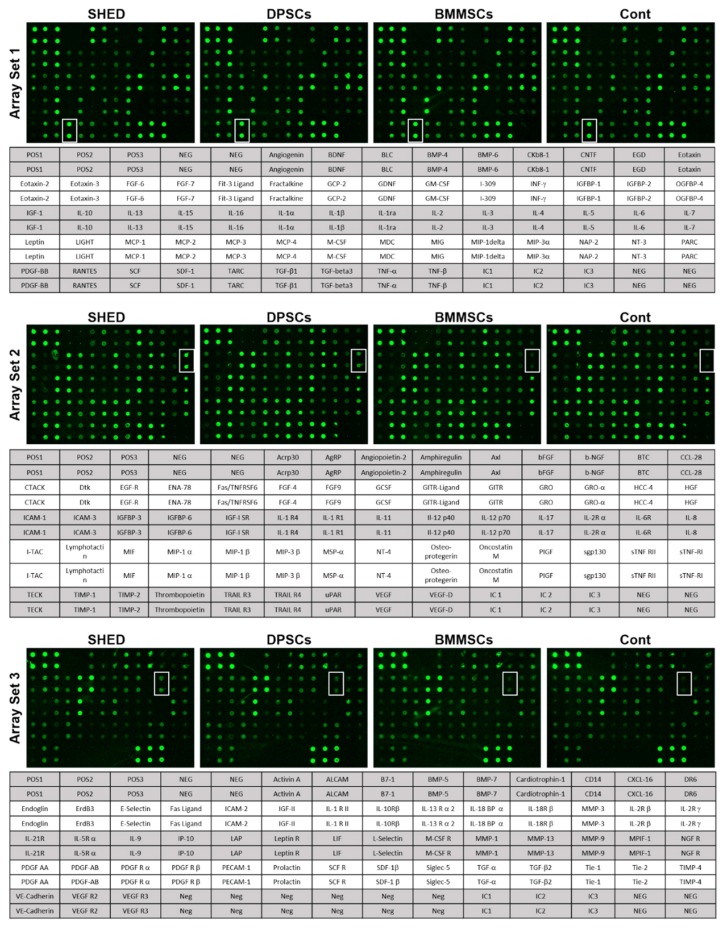
Images of the cytokine array blots probed with SHED, DPSCs, BMMSCs, and Dulbecco’s modified Eagle medium (DMEM) control samples. Each blot represents immunoreactive staining against the respective antibodies. The blots marked with a white box are the cytokines, stromal cell derived factor 1 (SDF-1)1 (Array Set 1), hepatocyte growth factor (HGF) (Array Set 2), and matrix metalloproteinase-3 (MMP-3) (Array Set 3) that were significantly up-regulated in SHEDs compared to BMMSCs. POS, positive control; NEG, negative control.

**Figure 3:**
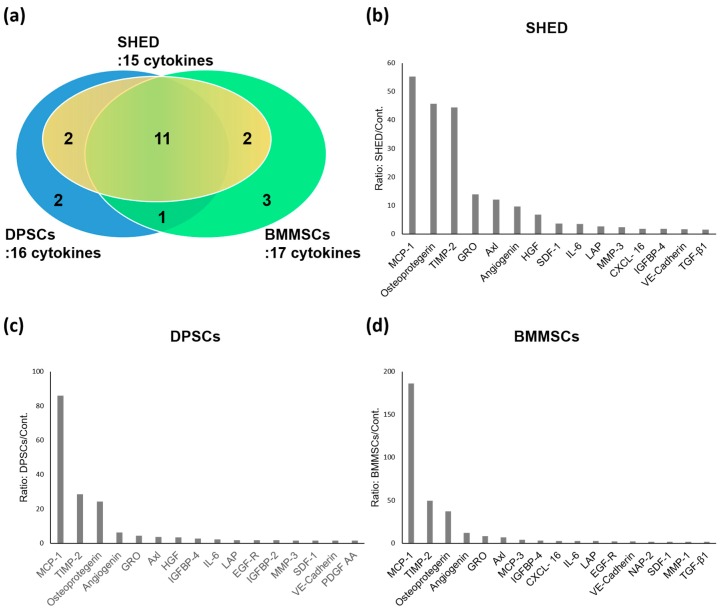
(**a**) Summary of the cytokines that were secreted from SHED, DPSCs, and BMMSCs. Cytokines expressed in SHED (**b**), DPSCs (**c**), and BMMSCs (**d**) at levels > 1.5-fold those in the control. A ≥ 1.5-fold increase is the threshold for a significant difference in expression according to the manufacturer’s instructions.

The cytokine antibody array analysis was used to identify the cytokines that were secreted from SHED, DPSCs, and BMMSCs (Figure 2). In total, 174 human cytokines were analyzed, and 1.5-fold or more expressed cytokines in SHED, DPSCs, and BMMSCs compared to the Dulbecco’s modified Eagle medium (DMEM)-control are shown in Figure 3. The cytokines that expressed lower levels than in the DMEM-control were eliminated in these analyses. These results indicated that there were 15, 16, and 17 cytokines with an increased expression of 1.5-fold or more in SHED, DPSCs, and BMMSCs compared to the control. Among these cytokines, 11 proteins, including monocyte chemoattractant protein-1 (MCP-1), osteoprotegerin, tissue inhibitor of metalloproteinase-2 (TIMP-2), growth regulated oncogene (GRO), Axl, angiogenin, stromal cell derived factor 1 (SDF-1), interleukin-6 (IL-6), latency associated peptide (LAP), insulin-like growth factor binding protein-4 (IGFBP-4), and vascular endothelial cadherin (VE-cadherin) were secreted from SHED, DPSCs, and BMMSCs. Hepatocyte growth factor (HGF) and matrix metalloproteinase-3 (MMP-3) were specific to stem cells from the dental pulp of both deciduous and permanent teeth. MCP-3, neutrophil activating protein-2 (NAP-2), and matrix metalloproteinase-1 (MMP-1) were specific to BMMSCs. A summary of cytokines secreted from SHED, DPSCs, and BMMSCs is shown in Table 1.

### 2.3. Comparison of Cytokines Expression Levels of SHED, DPSCs, and BMMSCs

**Figure 4:**
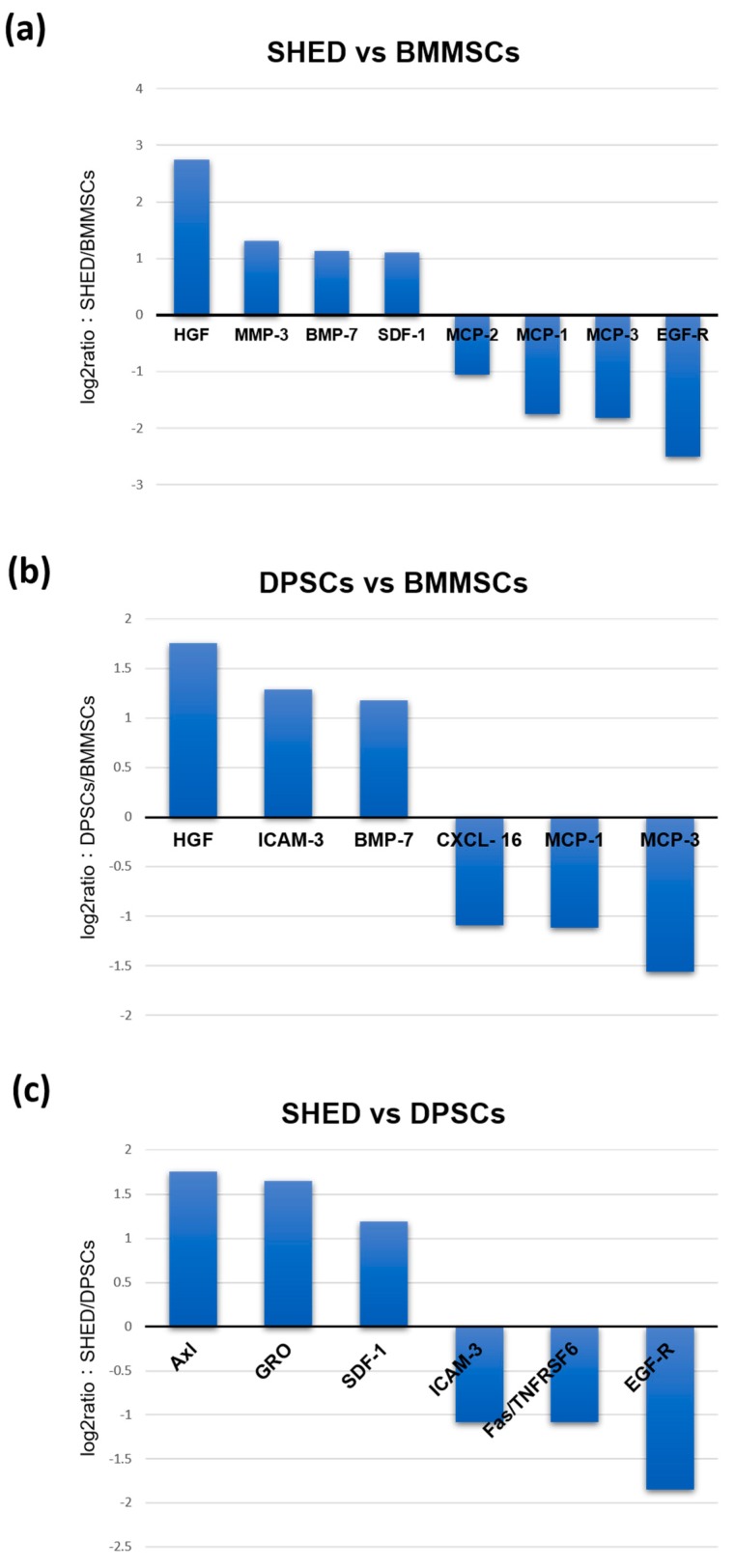
Comparative analyses of the secreted cytokines between SHED and BMMSCs (**a**), DPSCs and BMMSCs (**b**), and SHED and DPSCs (**c**).

Comparative analyses were performed among SHED, DPSCs, and BMMSCs. Difference factors at levels two-fold or higher are shown in Figure 4. The cytokines that expressed lower levels than the DMEM-control were eliminated in these analyses. The analyses between SHED and BMMSCs revealed that HGF, MMP-3, bone morphogenetic proteins-7 (BMP-7), and SDF-1 were expressed 6.7-, 2.5-, 2.2-, and 2.1-fold more strongly, respectively, in SHED (Figure 4). HGF and BMP-7 were also expressed 3.4-, and 2.3-fold higher in DPSCs than in BMMSCs, respectively. MCP-1 and MCP-3 were expressed more strongly in BMMSCs compared to both SHED and DPSCs. The SHED secretome contained higher concentrations of Axl (3.4-fold), GRO (3.1-fold), and SDF-1 (2.3-fold) than DPSCs.

### 2.4. Enzyme-Linked Immunosorbent Assay

**Figure 5:**
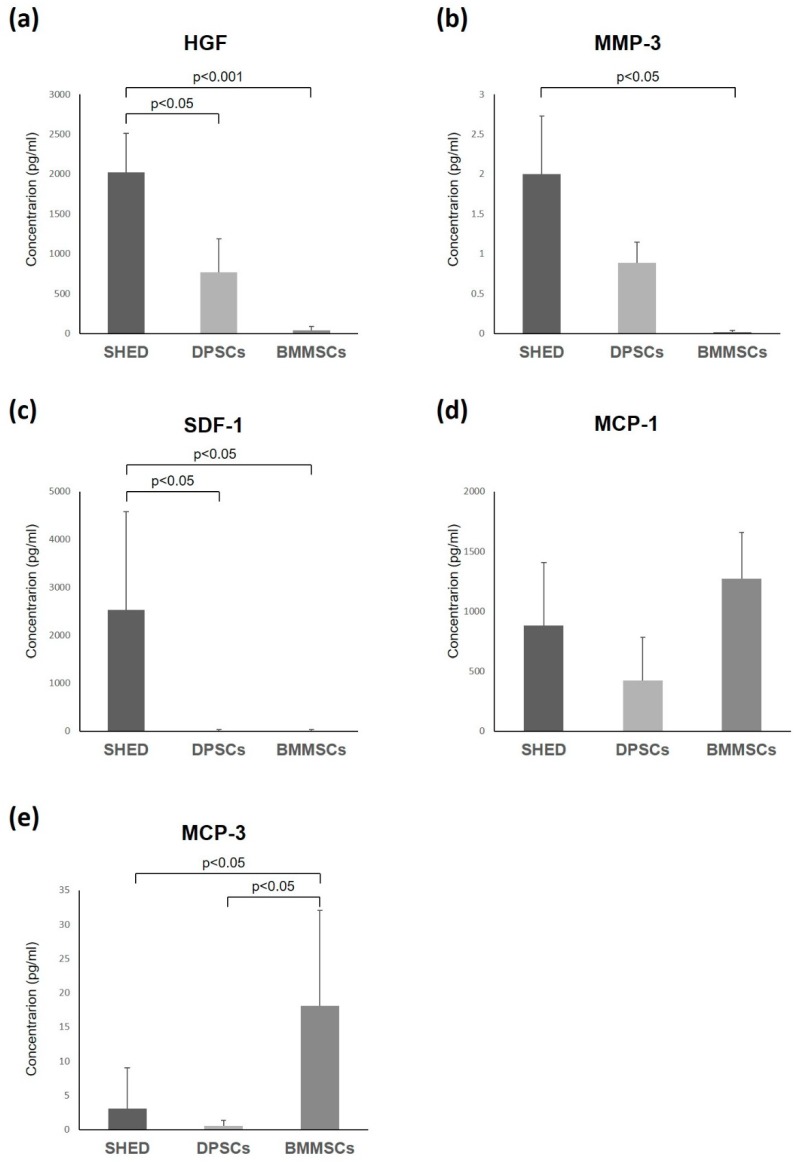
Quantitative analyses of the cytokines from SHED, DPSCs, and BMMSCs using enzyme-linked immunosorbent assay of HGF (**a**), MMP-3 (**b**), SDF-1 (**c**), MCP-1 (**d**), and MCP-3 (**e**). Bar: standard deviation.

To confirm the protein levels of cytokine expression, the expression patterns of related cytokines were validated using an enzyme-linked immunosorbent assay (ELISA) (Figure 5). The ELISA data revealed that the secretion of HGF, MMP-3, and SDF-1 were significantly higher in SHED than in BMMSCs. In addition, HGF and SDF-1 had a significantly higher expression in SHED than in DPSCs. MCP-1 and MCP-3 were expressed more strongly in BMMSCs than in both SHED and DPSCs. The expression level of MCP-3 in BMMSCs was significantly higher than that in SHED and DPSCs (Figure 3, Figure 4 and Figure 5). These results were consistent with the cytokine antibody array analysis results.

## 3. Discussion

Regenerative medicine is used to replace and repair damaged or diseased cells, tissues and organs to restore their normal functions with stem cells and related factors. The possibility to use endogenous and exogenous stem cells for tissue repair has emerged in the last 20 years [17]. The ultimate goal in the stem cell field and stem cell transplantation approaches is to find a way to translate our knowledge on stem cell biology into therapeutic applications for regenerative medicine. The need to overcome the drawbacks associated with the necessity of manipulating the cells and tissues before stem cell grafts has led to the development of new strategies to achieve tissue recovery and repair [18]. These grafting strategies require the exogenous activation of stem cells, endogenous re-activation, and the application of various cytokines and growth factors. These cytokines were originally identified in stem cell cultures and stem cells were activated in vivo [19]. In this study, we investigated the cytokines secreted from mesenchymal stem cells such as SHED, DPSCs, and BMMSCs for functional cell-based therapy to develop a new strategy. Out results indicated that SHED, DPSCs, and BMMSCs secreted common cytokines, such as chemokines; MCP-1, GRO, and SDF-1, inflammatory cytokines; osteoprotegerin, TIMP-2, IL-6, and LAP, and growth factors; angiogenin and insulin-like growth factor-binding protein-4 (IGFBP-2). However, comparative analyses among these stem cells revealed that they were widely different from each other in their expressed cytokine levels.

Quantitative analyses of the cytokines indicated that HGF was significantly higher in SHED than in DPSCs and BMMSCs. HGF was originally identified as a potent mitogen for mature hepatocytes. HGF also stimulates the proliferation and proteoglycan synthesis of some mesenchymal cells such as chondrocytes [20]. It has been indicated that HGF can also stimulate the proliferation and differentiation of progenitor cells [21]. In our previous study, the proliferation rate of SHED was significantly higher than that of DPSCs and BMMSCs. This result might be related to the HGF and the paracrine effect. That is, the expression of the HGF receptor is found mainly in epithelial cells, suggesting that HGF acts in a paracrine fashion to mediate interactions between epithelial and stromal cells during development and in normal tissue maintenance [22]. Previous studies also reported that HGF was more common in the conditioned medium of SHED than in that of BMMSCs and was involved in the protection of the heart from ischemic injury and the resolution of liver fibrosis [23,24]. In addition, HGF has gained attention as a strong neurotrophic factor in the central nervous system. Phase I/II clinical trials were conducted using rhHGF for acute cervical spinal cord injury (SCI) [25]. The introduction of exogenous HGF into the spinal cord by injecting an HGF-expressing herpes simplex virus (HSV) vector significantly increased the survival of neurons and oligodendrocytes, as well as increased angiogenesis and axonal regeneration, to reduce the area of damage and promote motor function of the hind limbs after SCI [26]. Therefore, the SHED secretome may be a promising source for cell-based SCI treatment. Our results imply that SHED might be better stem cells sources for systemic diseases such as myocardial infarction, liver fibrosis, and SCI than DPSCs and BMMSCs.

Matrix metalloproteinases (MMPs) are members of the metzincin group of proteases that mainly function in the breakdown of the extracellular matrix (ECM) [27]. They play an important role in many normal physiological processes such as embryonic development, morphogenesis, angiogenesis, reproduction, and tissue remodeling [28,29]. They also participate in many pathological processes such as arthritis, cancer, and cardiovascular disease [30]. MMPs may also affect bioactive molecules on the cell surface and modulate various cellular and signaling pathways. Alterations in MMP expression and activity occur in normal biological processes during pregnancy and wound healing [27]. MMP-3, a member of the MMP family, is reported to inactivate proinflammatory mediators, enhance the clearance of inflammatory cells, and regulate inflammatory conditions [31,32]. MMP-3 is well known as a secretory endopeptidase that degrades the ECM [33]. Previous studies indicated that MMP-3 may be involved in not only the physiological matrix turnover but also the pathological destruction of tissue and wound healing. [34,35]. In this study, the secretion of MMP-3 in SHED was significantly higher than that in BMMSCs. MMP-3 has been reported to be expressed in healthy dental pulp tissue and is involved in remodeling of the dentin matrix, suggesting that it may be related to the maintenance of pulpal homeostasis [35]. Moreover, it was reported that MMP-3 has anti-inflammatory effects in pulpal inflammation and plays a critical role in the angiogenesis and pulp wound healing of injured pulp tissue [35,36]. Our previous study indicated that SHED enhanced wound healing [37]. MMP-3 secreted from SHED may be involved in the wound healing process and could be a useful agent for clinical application.

SHED, DPSCs, and BMMSCs secreted various kinds of chemokines (Table 1). SDF-1 is a member of the CXC chemokine subfamily. SDF-1 binds to the CXC receptor 4 (CXCR4) on the cell surface of the responsive cells. It plays a role in the recruitment, migration, and differentiation of stem/progenitor cells including hematopoietic/endothelial progenitor cells and mesenchymal stem cells [38]. SDF-1 has been used for the tissue engineering and regeneration of various tissues such as myocardia [39], liver [40], nerve [41], cartilage [42], and bones [43]. SDF-1 was also shown to promote the odontogenic differentiation of human dental pulp cells [44], and is related to pulp regeneration [45]. In this study, SDF-1 were more expressed in SHED than in DPSCs and BMMSCs (Figure 3, Figure 4 and Figure 5). Therefore, the secretome from SHED may be used by endogenous stem cells or cytokine-mediated activation and/or mobilization of stem cells as treatment for various diseases and it may be useful for pulp regeneration.

On the other hand, MCP-1 is one of the key chemokines that regulates the migration and infiltration of monocytes/macrophages and provides essential signaling for normal bone healing [46]. MCP-1 also is known to contribute to the proinflammatory M1 response by recruiting macrophages to inflammation sites [47]. MCP-3 is another signaling pathway used to recruit progenitors from systemic circulation [48] and pro-inflammatory pathways, activating leukocytes by binding to several chemokine receptors [49]. MCP-1 and MCP-3 are MSCs homing factors and recruit MSCs to sites of injured tissue while also improving cardiac remodeling [46,50]. In this study, MCP-1 was secreted at high levels in all SHED, DPSCs, and BMMSCs. MCP-1 and MCP-3 were expressed more strongly in BMMSCs compared to both SHED and DPSCs (Figure 3, Figure 4 and Figure 5). MCP-1 may be related to MSCs’ character, which exhibits unique immunoregulatory properties that contribute to tissue-repair; MCP-1 may also be a novel, potent therapeutic target for functional cell-based therapy. Moreover, the SHED secretome contained higher concentrations of Axl (3.4-fold) than DPSCs (Figure 4). Our previous studies indicated that SHED had significantly higher proliferation ability than DPSCs and BMMSCs. The comparative analysis of gene expression between SHED and DPSCs indicated that the cell proliferation network was accelerated in SHED than in DPSCs [13]. The role of Axl is related to extending the mediation of processes such as proliferation, migration, and adhesion in both normal and disease settings [51]. Therefore, Axl might be also related to the high proliferation ability of SHED.

Taken together, our results indicate that stem cells originating from exfoliated deciduous teeth dental pulp, permanent teeth dental pulp, and bone marrow have different cytokine profiles, despite being defined as MSCs, thereby indicating that they have identifying characteristics. The variety of cytokine expression levels may influence their tissue regeneration abilities. It might be important to select stem cells that are most suitable for the treatment of various diseases by determining their own features. Although further studies are required to examine the details of each cytokine function secreted from SHED, DPSCs, and BMMSCs, the cytokine secretion profile of this study will be useful for regenerative medicine. In conclusion, on the basis of this distinctive analysis of cytokine secreted from exfoliated deciduous teeth, dental pulp derived, and bone marrow derived mesenchymal stem cells, this study could extend, play a role in, and provide a new therapeutic paradigm shift for functional cell-based therapies in regenerative medicine.

## 4. Materials and Methods

### 4.1. Subjects and Cell Cultures

Human dental pulp tissues were obtained from clinically healthy extracted deciduous teeth and permanent teeth from individuals. SHED and DPSCs were isolated and cultured as previously described [8]. Briefly, pulp was gently removed and digested in a solution of 3 mg/mL collagenase type I and 4 mg/mL dispase at 37 °C. The cells were cultured in Dulbecco’s modified Eagle medium (DMEM; GIBCO, Rockville, MD, USA) containing 20% mesenchymal cell growth supplement (Lonza Inc., Walkersville, MD, USA) and antibiotics (100 U/mL penicillin, 100 mg/mL streptomycin, and 0.25 mg/mL amphotericin B; GIBCO) at 37 °C under 5% CO_2_. Human BMMSCs were isolated according to the reported method [14]. Briefly, BMMSCs were cultured in DMEM supplemented with 10% mesenchymal cell growth supplement (Lonza Inc.) and antibiotics (100 U/mL penicillin, 100 mg/mL streptomycin, and 0.25 mg/mL amphotericin B; GIBCO) at 37 °C under 5% CO_2_. The study was conducted in accordance with the Declaration of Helsinki and ethical approval was obtained from the ethics committee. All participants provided written informed consent as previously described [14].

### 4.2. Flow Cytometry Analyses

Flow cytometry analysis was performed as described previously [8]. In brief, cultured cells were trypsinized, centrifuged, washed with phosphate-buffered saline (PBS), and incubated for 45 min at 4 °C with specific antibodies. Phycoerythrin-conjugated mouse antibodies against human CD73 (BD Pharmingen, San Diego, CA, USA), allophycocyanin-conjugated mouse antibodies against human CD34 (BD Pharmingen), fluorescein isothiocyanate-conjugated mouse antibodies against human CD105 (BD Pharmingen), and peridinin-chlorophyll-protein-conjugated mouse antibodies against human CD45 (BD Pharmingen) were used to analyze specific surface antigens. Cell fluorescence was evaluated by flow cytometry using FACS Calibur (BD Pharmingen).

### 4.3. Cytokine Antibody Array Analysis

The human cytokine antibody array analysis was performed using a RayBio^®^ Human Cytokine Antibody Array G Series 2000 (RayBiotech Inc. Norcross, GA, USA) at Filgen according to the manufacturer’s instructions. Briefly, SHEDs, DPSCs, and BMMSCs were cultured until they reached 80% confluence and the culture cells were washed with PBS and incubated with DMEM containing 0.2% FBS. After 48 h, the conditioned medium was collected and used in the cytokine antibody array analysis. DMEM containing 0.2% FBS as used for the negative controls. Array membranes were incubated for 30 min at room temperature in a blocking buffer. Arrays with sample were incubated at room temperature for 2 h and then washed with washing buffer. After being incubated with biotin-conjugated antibody, membranes were washed and incubated with fluorescent dye-conjugated streptavidin for 1 h. All scans were performed in duplicate. Signal intensities were quantified with the Array-Pro Analyzer^®^ Ver.4.5 (Mediacy bernetics, Inc., Bethesda, MD, USA), and the intensities of the same cytokine from different samples were compared.

### 4.4. Enzyme-Linked Immunosorbent Assay

For the quantitative determination of human cytokine concentrations, enzyme-linked immunosorbent assay was performed using The Quantikine Human HGF Immunoassay (R&D Systems, Inc. Minneapolis, MN, USA), Human CCL2/MCP-1 Immunoassay (R&D Systems, Inc.), Human CCL7/MCP-3 Immunoassay (R&D Systems, Inc.), Human Total MMP-3 Immunoassay (R&D Systems, Inc.), and Human CXCL12/SDF-1 Immunoassay (R&D Systems, Inc.) according to the manufacturer’s instructions. The ELISA data were obtained from four independent SHED, DPSCs, and BMMSCs, respectively, each of which was performed four times.

### 4.5. Statistical Analysis

All data are expressed as the mean ± SEM. The statistical analyses were performed using the SigmaPlot software 12.3. Differences among the experimental groups were examined by one-way analysis of variance (ANOVA) tests using Tukey’s honest significant difference test. A *p* value less than 0.05 was considered to be statistically significant.

## Figures and Tables

**Table 1:** Summary of cytokines secreted by SHEDs, DPSCs, and BMMSCs. (++; ≥ 2 fold expression to control, +; ≥1.5 fold expression to control, ―; < 1.5 fold expression to control).

Cytokines		SHEDs	DPSCs	BMMSCs
Growth factors	Angiogenin	++	++	++
	HGF	++	++	―
	TGF-β1	+	―	+
	EGF-R	―	+	++
	IGFBP-2	―	+	―
	IGFBP-4	+	++	++
	PDGF AA	―	+	―
Chemokines	MCP-1	++	++	++
	MCP-3	―	―	++
	GRO	++	++	++
	SDF-1	++	+	+
	CXCL- 16	+	―	++
	NAP-2	―	―	+
Inflammatory cytokines	Osteoprotegerin	++	++	++
	TIMP-2	++	++	++
	IL-6	++	++	++
	LAP	++	+	++
Other cytokines	Axl	++	++	++
	VE-Cadherin	+	+	+
	MMP-1	―	―	+
	MMP-3	++	+	―

Abbreviations: HGF, hepatocyte growth factor; TGF-β1, transforming growth factor beta 1; EGF-R, epidermal growth factor receptor; IGFBP-2, insulin-like growth factor binding protein-2; IGFBP-4, insulin-like growth factor binding protein-4; PDGF AA, Platelet derived growth factor AA; MCP-1, monocyte chemoattractant protein-1; MCP-3, monocyte chemoattractant protein-3; GRO, growth regulated oncogene; SDF-1, stromal cell derived factor 1; CXCL-16, C-X-C motif ligand-16; NAP-2, Neutrophil Activating Protein-2; TIMP-2, tissue inhibitor of metalloproteinase-2; IL-6, Interleukin-6; LAP, latency associated peptide; VE-Cadherin, vascular endothelial cadherin; MMP-1, matrix metalloproteinase-1; MMP-3, matrix metalloproteinase-3.

## Abbreviations

MSCs	mesenchymal stem/stromal cells
SHED	stem cells from exfoliated deciduous teeth
DPSCs	dental pulp stem cells
BMMSCs	bone marrow-derived mesenchymal stem cells
MCP-1	monocyte chemoattractant protein-1
TIMP-2	tissue inhibitor of metalloproteinase-2
GRO	growth regulated oncogene
SDF-1	stromal cell derived factor 1
IL-6	interleukin-6
LAP	latency associated peptide
IGFBP-4	insulin-like growth factor binding protein-4
VE-Cadherin	vascular endothelial cadherin
HGF	hepatocyte growth factor
MMP-3	matrix metalloproteinase-3
MCP-3	monocyte chemoattractant protein-1
NAP-2	neutrophil activating protein-2
MMP-1	matrix metalloproteinase-1
TGF-β1	Transforming growth factor beta 1
EGF-R	epidermal growth factor receptor
IGFBP-2	insulin-like growth factor binding protein-2
PDGF AA	platelet derived growth factor AA
CXCL-16	C-X-C motif ligand-16
ELISA	enzyme-linked immunosorbent assay
SCI	spinal cord injury
MMPs	matrix metalloproteinases
ECM	extracellular matrix
CXCR4	CXC receptor 4
DMEM	Dulbecco’s modified Eagle medium
PBS	phosphate-buffered saline

## References

[1] Pittenger M.F., Mackay A.M., Beck C.B., Jaiswal R.K., Douglas R., Mosca J.D., Moorman M.A., Simonetti D.W., Craiq S., Marshak D.R. (1999). Multilineage potential of adult human mesenchymal stem cells. Science.

[2] Yamada Y., Nakamura-Yamada S., Kusano K., Baba S. (2019). Clinical Potential and Current Progress of Dental Pulp Stem Cells for Various Systemic Diseases in Regenerative Medicine: A Concise Review. Int. J. Mol. Sci..

[3] Zuk P.A., Zhu M., Ashjian P., De Ugarte D.A., Huang J.I., Mizuno H., Alfonso Z.C., Fraser J.K., Benhaim P., Hedrick M.H. (2002). Human adipose tissue is a source of multipotent stem cells. Mol. Biol. Cell.

[4] Young H.E., Steele T.A., Bray R.A., Hudson J., Floyd J.A., Hawkins K., Thomas K., Austin T., Edwards C., Cuzzourt J. (2001). Human reserve pluripotent mesenchymal stem cells are present in the connective tissues of skeletal muscle and dermis derived from fetal, adult, and geriatric donors. Anat. Rec..

[5] Wang H.S., Hung S.C., Peng S.T., Huang C.C., Wei H.M., Guo Y.J., Fu Y.S., Lai M.C., Chen C.C. (2004). Mesenchymal stem cells in the Wharton’s jelly of the human umbilical cord. Stem Cells.

[6] Gronthos S., Mankani M., Brahim J., Robey P.G., Shi S. (2000). Postnatal human dental pulp stem cells (DPSCs) in vitro and in vivo. Proc. Natl. Acad. Sci. USA.

[7] Miura M., Gronthos S., Zhao M., Lu B., Fisher L.W., Robey P.G., Shi S. (2003). SHED: Stem cells from human exfoliated deciduous teeth. Proc. Natl. Acad. Sci. USA.

[8] Yamada Y., Nakamura S., Ito K., Sugito T., Yoshimi R., Nagasaka T., Ueda M. (2010). A feasibility of useful cell-based therapy by bone regeneration with deciduous tooth stem cells, dental pulp stem cells, or bone-marrow-derived mesenchymal stem cells for clinical study using tissue engineering technology. Tissue Eng. Part A.

[9] Yamada Y., Ito K., Nakamura S., Ueda M., Nagasaka T. (2011). Promising cell-based therapy for bone regeneration using stem cells from deciduous teeth, dental pulp, and bone marrow. Cell Transplant..

[10] Dominici M., le Blanc K., Mueller I., Slaper-Cortenbach I., Marini F., Krause D., Deans R., Keating A., Prockop D., Horwitz E. (2006). Minimal criteria for defining multipotent mesenchymal stromal cells. The International Society for Cellular Therapy position statement. Cytotherapy.

[11] Eleuteri S., Fierabracci A. (2019). Insights into the Secretome of Mesenchymal Stem Cells and Its Potential Applications. Int. J. Mol. Sci..

[12] Yamada Y., Fujimoto A., Ito A., Yoshimi R., Ueda M. (2006). Cluster analysis and gene expression profiles: A cDNA microarray system-based comparison between human dental pulp stem cells (hDPSCs) and human mesenchymal stem cells (hMSCs) for tissue engineering cell therapy. Biomaterials.

[13] Nakamura S., Yamada Y., Katagiri W., Sugito T., Ito K., Ueda M. (2009). Stem cell proliferation pathways comparison between human exfoliated deciduous teeth and dental pulp stem cells by gene expression profile from promising dental pulp. J. Endod..

[14] Hara K., Yamada Y., Nakamura S., Umemura E., Ito K., Ueda M. (2011). Potential characteristics of stem cells from human exfoliated deciduous teeth compared with bone marrow-derived mesenchymal stem cells for mineralized tissue-forming cell biology. J. Endod..

[15] Yamada Y., Nakaumura S., Ito K., Umemura E., Hara K., Nagasaka T., Abe A., Baba S., Furuichi Y., Izumi Y. (2013). Injectable Bone Tissue Engineering Using Expanded Mesenchymal Stem Cells. Stem Cells.

[16] Phinney D.G., Pittenger M.F. (2017). Concise Review: MSC—Derived Exosomes for Cell-Free Therapy. Stem Cells.

[17] Mason C., Dunnill P. (2008). A brief definition of regenerative medicine. Regen. Med..

[18] Su X., Paris M., Gi Y.J., Tsai K.Y., Cho M.S., Lin Y.L., Biernaskie J.A., Sinha S., Prives C., Pevny L.H. (2009). TAp63 prevents premature aging by promoting adult stem cell maintenance. Cell Stem Cell.

[19] Miller F.D., Kaplan D.R. (2012). Mobilizing endogenous stem cells for repair and regeneration: Are we there yet?. Cell Stem Cell.

[20] Russell W.E., McGowan J.A., Bucher N.L. (1984). Partial characterization of a hepatocyte growth factor from rat platelets. J. Cell. Physiol..

[21] Graziani A., Gramaglia D., dalla Zonca P., Comoglio P.M. (1993). Hepatocyte growth factor/scatter factor stimulates the Ras-guanine nucleotide exchanger. J. Biol. Chem..

[22] Zarnegar R., Michalopoulos G.K. (1995). The many faces of hepatocyte growth factor: From hepatopoiesis to hematopoiesis. J. Cell Biol..

[23] Yamaguchi S., Shibata R., Yamamoto N., Nishikawa M., Hibi H., Tanigawa T., Ueda M., Murohara T., Yamamoto A. (2015). Dental pulp-derived stem cell conditioned medium reduces cardiac injury following ischemia-reperfusion. Sci. Rep..

[24] Hirata M., Ishigami M., Matsushita Y., Ito T., Hattori H., Hibi H., Goto H., Ueda M., Yamamoto A. (2016). Multifaceted Therapeutic Benefits of Factors Derived From Dental Pulp Stem Cells for Mouse Liver Fibrosis. Stem Cells Transl. Med..

[25] Kitamura K., Nagoshi N., Tsuji O., Matsumoto M., Okano H., Nakamura M. (2019). Application of Hepatocyte growth factor for acute spinal cord injury: The road from basic studies to human treatnment. Int. J. Mol. Sci..

[26] Kitamura K., Iwanami A., Nakamura M., Yamane J., Watanabe K., Suzuki Y., Miyazawa D., Shibata S., Funakoshi H., Miyatake S. (2007). Hepatocyte growth factor promotes endogenous repair and functional recovery after spinal cord injury. J. Neurosci. Res..

[27] Cui N., Hu M., Khalil R.A. (2017). Biochemical and Biological Attributes of Matrix Metalloproteinases. Prog. Mol. Biol. Transl. Sci..

[28] Rundhaug J.E. (2005). Matrix metalloproteinases and angiogenesis. J. Cell Mol. Med..

[29] Nagase H., Woessner J.F. (1999). Matrix metalloproteinases. J. Biol. Chem..

[30] Fini M.E., Cook J.R., Mohan R., Brinckerhoft C.E., Parks W.C., Mecham R.P. (1998). Matrix Metalloproteinases.

[31] Page-McCaw A., Ewald A.J., Werb Z. (2007). Matrix metalloproteinases and the regulation of tissue remodelling. Nat. Rev. Mol. Cell Biol..

[32] Parks W.C., Wilson C.L., López-Boado Y.S. (2004). Matrix metalloproteinases as modulators of inflammation and innate immunity. Nat. Rev. Immunol..

[33] Eguchi T., Kubota S., Kawata K., Mukudai Y., Uehara J., Ohgawara T., Ibaragi S., Sasaki A., Kuboki T., Takigawa M. (2008). Novel transcription-factor-like function of human matrix metalloproteinase 3 regulating the CTGF/CCN2 gene. Mol. Cell. Biol..

[34] Haro H., Crawford H.C., Fingleton B., MacDougall J.R., Shinomiya K., Spengler D.M., Matrisian L.M. (2000). Matrix metalloproteinase-3-dependent generation of a macrophage chemoattractant in a model of herniated disc resorption. J. Clin. Investig..

[35] Takimoto K., Kawashima N., Suzuki N., Koizumi Y., Yamamoto M., Nakashima M., Suda H. (2014). Down-regulation of inflammatory mediator synthesis and infiltration of inflammatory cells by MMP-3 in experimentally induced rat pulpitis. J. Endod..

[36] Zheng L., Amano K., Iohara K., Ito M., Imabayashi K., Into T., Matsushita K., Nakamura H., Nakashima M. (2009). Matrix metalloproteinase-3 accelerates wound healing following dental pulp injury. Am. J. Pathol..

[37] Nishino Y., Yamada Y., Ebisawa K., Nakamura S., Okabe K., Umemura E., Hara K., Ueda M. (2011). Stem cells from human exfoliated deciduous teeth (SHED) enhance wound healing and the possibility of novel cell therapy. Cytotherapy.

[38] Liekens S., Schols D., Hatse S. (2010). CXCL12-CXCR4 axis in angiogenesis, metastasis and stem cell mobilization. Curr. Pharm. Des..

[39] Song M., Jang H., Lee J., Kim J.H., Kim S.H., Sun K., Park Y. (2014). Regeneration of chronic myocardial infarction by injectable hydrogels containing stem cell homing factor SDF-1 and angiogenic peptide Ac-SDKP. Biomaterials.

[40] Ding B.S., Cao Z., Lis R., Nolan D.J., Guo P., Simons M., Penfold M.E., Shido K., Rabbany S.Y., Rafii S. (2014). Divergent angiocrine signals from vascular niche balance liver regeneration and fibrosis. Nature.

[41] Heskamp A., Leibinger M., Andreadaki A., Gobrecht P., Diekmann H., Fischer D. (2013). CXCL12/SDF-1 facilitates optic nerve regeneration. Neurobiol. Dis..

[42] Zhang W., Chen J., Tao J., Jiang Y., Hu C., Huang L., Ji J., Ouyang H.W. (2013). The use of type 1 collagen scaffold containing stromal cell-derived factor-1 to create a matrix environment conducive to partial-thickness cartilage defects repair. Biomaterials.

[43] Ji W., Yang F., Ma J., Bouma M.J., Boerman O.C., Chen Z., van den Beucken J.J., Jansen J.A. (2013). Incorporation of stromal cell-derived factor-1alpha in PCL/gelatin electrospun membranes for guided bone regeneration. Biomaterials.

[44] Kim D.S., Kim Y.S., Bae W.J., Lee H.J., Chang S.W., Kim W.S., Kim E.C. (2014). The role of SDF-1 and CXCR4 on odontoblastic differentiation in human dental pulp cells. Int. Endod. J..

[45] Iohara K., Imabayashi K., Ishizaka R., Watanabe A., Nabekura J., Ito M., Matsushita K., Nakamura H., Nakashima M. (2011). Complete pulp regeneration after pulpectomy by transplantation of CD105+ stem cells with stromal cell-derived factor-1. Tissue Eng. Part A.

[46] Deshmane S.L., Kremlev S., Amini S., Sawaya B.E. (2009). Monocyte chemoattractant protein-1 (MCP-1): An overview. J. Interferon Cytokine Res..

[47] Tsou C.L., Peters W., Si Y., Slaymaker S., Aslanian A.M., Weisberg S.P., Mack M., Charo I.F. (2007). Critical roles for CCR2 and MCP-3 in monocyte mobilization from bone marrow and recruitment to inflammatory sites. J. Clin. Investig..

[48] Schenk S., Mal N., Finan A., Zhang M., Kiedrowski M., Popovic Z., McCarthy P.M., Penn M.S. (2007). Monocyte chemotactic protein-3 is a myocardial mesenchymal stem cell homing factor. Stem Cells.

[49] Menten P., Wuyts A., Van Damme J. (2001). Monocyte chemotactic protein-3. Eur. Cytokine Netw..

[50] Shinohara K., Greenfield S., Pan H., Vasanji A., Kumagai K., Midura R.J., Kiedrowski M., Penn M.S., Muschler G.F. (2011). Stromal cell-derived factor-1 and monocyte chemotactic protein-3 improve recruitment of osteogenic cells into sites of musculoskeletal repair. J. Orthop. Res..

[51] Axelrod H., Pienta K.J. (2014). Axl as a mediator of cellular growth and survival. Oncotarget.

